# Case Report: A case of influenza A infection-associated stiff person spectrum disorder with favorable outcome

**DOI:** 10.3389/fimmu.2025.1673538

**Published:** 2025-10-14

**Authors:** Xiaoqian Liu, Meifang Lei, Mengna Tian, Yanfen Zhang, Xiaoli Yu, Dong Li

**Affiliations:** ^1^ Department of Pediatric Neurology, Tianjin Children’s Hospital/Children’s Hospital, Tianjin University, Tianjin, China; ^2^ Department of Neuroelectrophysiology, Pediatric Neurology, Tianjin Children’s Hospital/Children’s Hospital, Tianjin University, Tianjin, China

**Keywords:** stiff person spectrum disorder, influenza A, autoimmunity, electromyography, immunotherapy

## Abstract

Influenza A (H1N1) virus infection has been associated with various immune-mediated neurological disorders. Previous studies have reported cases of stiff person spectrum disorder (SPSD) subsequent to viral infections. We report a case of a 4-year-old boy who developed SPSD following H1N1 infection. The patient primarily presented with axial and limb muscle rigidity, painful spasms, and exaggerated startle responses. Electromyography (EMG) revealed continuous synchronous discharges in agonist and antagonist muscles, consistent with the characteristic manifestations of stiff person syndrome (SPS), meanwhile, repetitive compound muscle action potentials (R-CMAPs) following stimulation of the median, common peroneal and tibial nerves were observed on electroneurography (ENG). Symptoms markedly improved following benzodiazepine therapy, and complete remission was achieved after combined treatment with IVIG and glucocorticoids.

## 1 Introduction

Stiff person spectrum disorder (SPSD) is a rare group of neuroimmunological disorders characterized by progressive rigidity and triggered painful spasms of the axial musculature. The clinical spectrum varies from classical stiff person syndrome (SPS), stiff limb (or leg) syndrome (SLS), to progressive encephalomyelitis with rigidity and myoclonus (PERM) (1, 2). Antibodies associated with SPSD include anti-GAD65 antibodies usually found in classic SPS, anti-glycine receptor (anti-GlyR) antibodies commonly found in PERM, antibodies against amphiphysin/dipeptidyl-peptidase-like protein(DPPX) in subsets, including paraneoplastic, etc. H1N1 virus infection is associated with seizures, influenza-associated encephalopathy or encephalitis (IAE) (3), acute disseminated encephalomyelitis (ADEM), Guillain-Barre syndrome (4). We report a case of a 4-year-old boy who developed SPSD following H1N1 infection. The patient primarily presented with axial and limb muscle rigidity, painful spasms, and exaggerated startle responses. Electromyography (EMG) revealed continuous synchronous discharges in agonist and antagonist muscles, consistent with the characteristic manifestations of stiff person syndrome (SPS). Symptoms remission was achieved after treatment with Intravenous Immunoglobulin (IVIG), glucocorticoids and benzodiazepine. SPSD is exceedingly rare in the pediatric population, early immunomodulatory treatment is critical for improving long-term neurological outcomes.

## 2 Case presentation

A 4-year-old boy was admitted to our hospital during the local H1N1 epidemic with a 7-day history of intermittent fever and 1-day history of pain in the right lower extremity, accompanied by abnormal gait. On the second day of admission, muscular hypertonia was observed in the extremities, neck muscles, and abdominal muscles, resulting in a posture characterized by flexion of the upper limbs and extension of the lower limbs. The patient demonstrated difficulty in sitting up, reduced facial expression, and a tense, restless mental state. External stimuli exacerbated rigidity, and somatosensory stimulation could trigger a startle-like response. However, the pharyngeal and laryngeal muscle groups were not involved, as evidenced by preserved speech and swallowing functions. The symptoms showed mild alleviation during sleep. Partial improvement was observed following diazepam administration.

The patient was the first child of non-consanguineous Chinese parents, born at full term with normal birth parameters. Age-appropriate psychomotor development was noted. He has no previous history of other illnesses or medication use. No family history of genetic disorders was reported.

During examination, the patient was alert and anxious, presented with a forced head position but was fully conscious and articulate. The patient displayed hyperreflexia and exaggerated startle response to noise, tactile (such as light touch), accompanied by pain. Repeated stimuli consistently elicited these startle reactions. General responsiveness was preserved, with no observed rash, cyanosis, jaundice, or herpetic lesions. Cranial nerve examination revealed no abnormalities. Lead-pipe rigidity was noted in both lower extremities, while the upper limbs exhibited a persistent flexed and rigid posture. Abdominal muscles rigidity was evident, presenting as a board-like rigidity with intact abdominal wall reflexes (+). Babinski’s sign was negative bilaterally. Though difficult to test, coordination appeared to be normal and a slight decline in muscle strength was detected. Nuchal rigidity was observed due to the markedly increased neck muscle tone.

**Figure 1:**
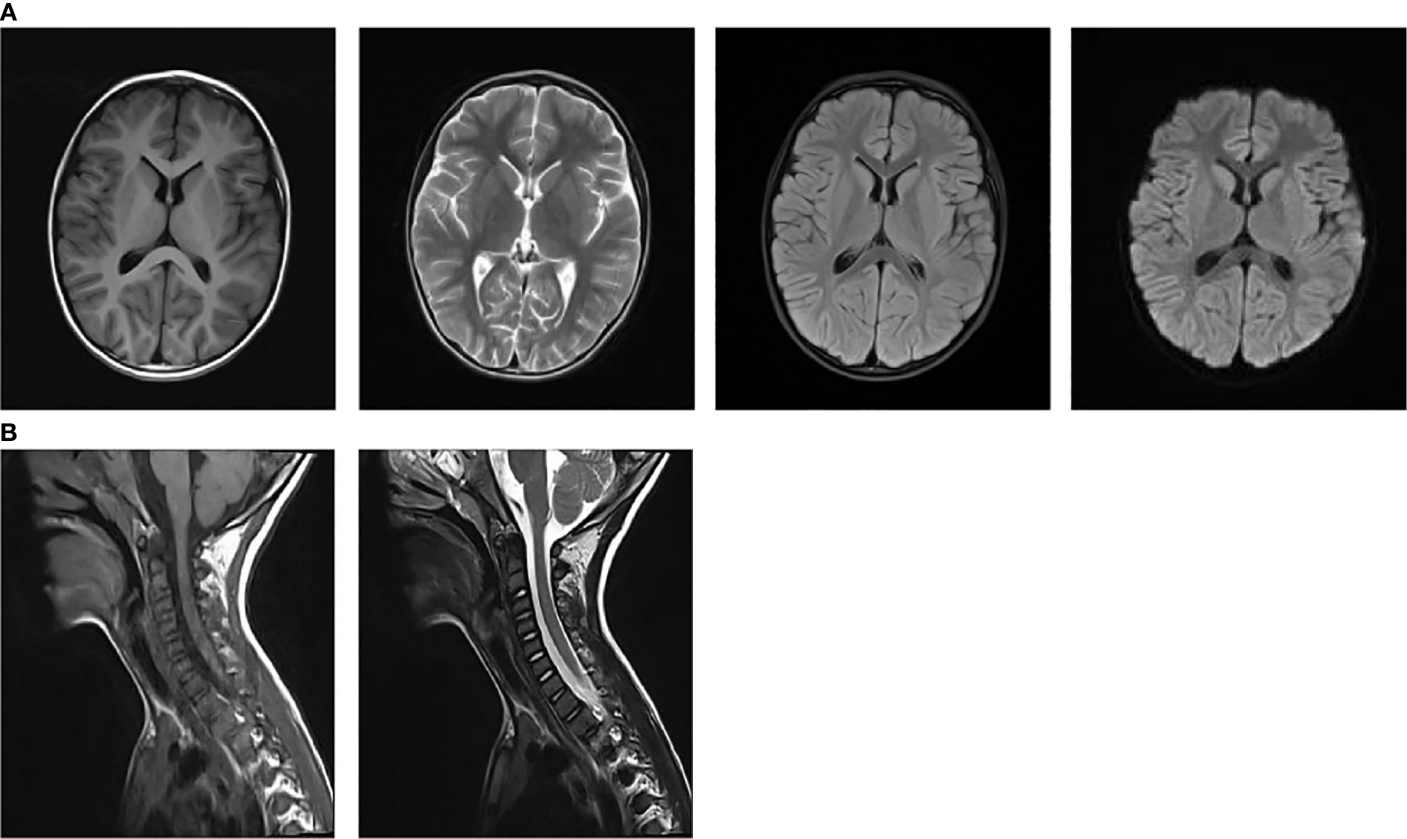
The head **(A)** and the cervical spinal cord **(B)** MRI examination was negative.

**Figure 2:**
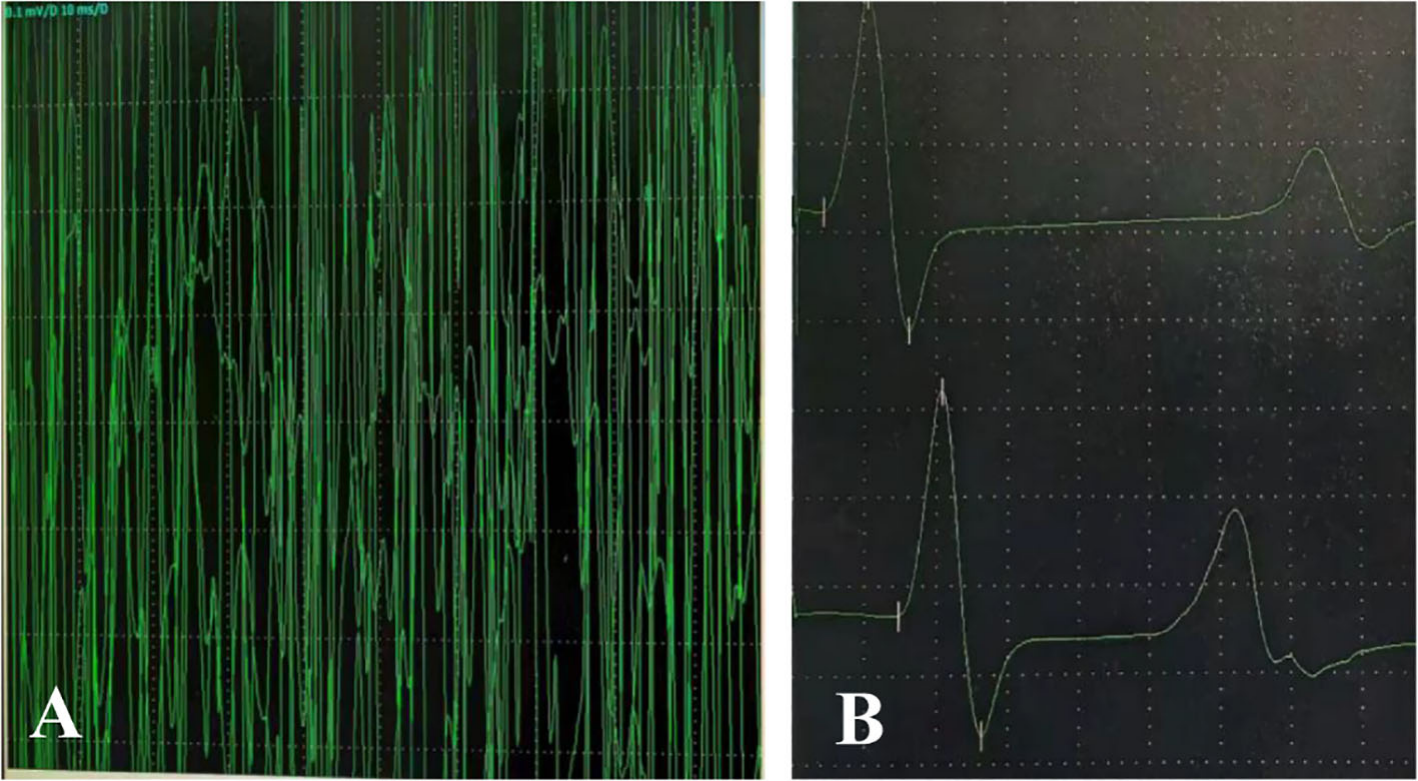
Continuous discharges were recorded in the gastrocnemius muscle (antagonist) during contraction of the tibialis anterior muscle (agonist) **(C)**. Upon electrical stimulation of the tibial nerve, repetitive compound muscle action potentials (R-CMAPs) with slightly reduced amplitude were observed after 20–50 milliseconds **(D)**.

On admission, nasopharyngeal swab test was positive for H1N1. Routine laboratory exams showed mild neutrophilic leukocytosis and raised inflammatory markers (PCT 0.25 ng/ml, n.v. <0.05; interleukin 6 109.05 pg/ml, n.v. <7.1; interleukin 10 49.1pg/ml, n.v. <10.6). Chest radiography revealed increased and disorganized bilateral lung markings. Routine and biochemical cerebrospinal fluid (CSF) analysis were normal. CSF microbiological analyses were negative. Diagnostic work-up for malignancy with abdominal and testicular ultrasonography was unremarkable. Antinuclear antibody (ANA) and anti-extractable nuclear antigen antibody (ENA) testing yielded negative results. Thyroid function and thyroid ultrasonography showed no abnormalities.1.5 T magnetic resonance imaging (MRI) of the brain and cervical spinal cord were unremarkable at 1 week after symptoms onset (**Figures 1A, B**). Electroencephalogram (EEG) with polygraphy excluded a cortical origin of myoclonus. Needle EMG showed continuous motor unit activity (CMUA) and co-activation of agonist and antagonist muscles with normal-appearing motor unit potentials (MUPs), particularly in the lower extremities (**Figure 2A**). R-CMAPs were recorded by stimulating the median, tibial, and common peroneal nerves for approximately 20–50 milliseconds (**Figure 2B**).

Antibodies were detected by indirect immunofluorescence test (IIFT), yielded negative results in both serum and CSF(NMDAR, AMPAR, LGI1, CASPR2, GABAAR, GABABR, IgLON5, DPPX, GlyRα1, mGluR5, D2R, Neurexin-3α, GAD65, KLHL11, AK5), and also paraneoplastic antibody panels(Hu, Yo, Ri, MA2, CV2, Amphiphysin, ANNA-3, Tr, PCA-2) in serum. The negative antibody results in this case may be attributable to several factors: undetected known antibodies, unidentified novel antibodies, cell-mediated immune mechanisms, or low-titer antibodies.

Differential diagnosis was systematically performed. The patient had no history of skin trauma and did not exhibit typical tetanus manifestations such as trismus or hydrophobia, and the effectiveness of benzodiazepine therapy further ruled out tetanus. Neuromyotonia is electrophysiologically characterized by spontaneous, continuous motor unit discharges of high firing frequencies (150–300 Hz), which were not observed in this case.

The patient presented with stiffness (axial or limb), episodic spasms triggered by noises, tactile stimuli, emotional stress, increased muscle tone (axial or limb), concurrent stiffness of abdominal muscles, co-contraction of agonist/antagonist muscles by EMG, after exclusion of other differential diagnoses, he met the diagnostic criteria for probable SPSD, with benzodiazepine responsiveness as supportive (5).

**Figure 3:**
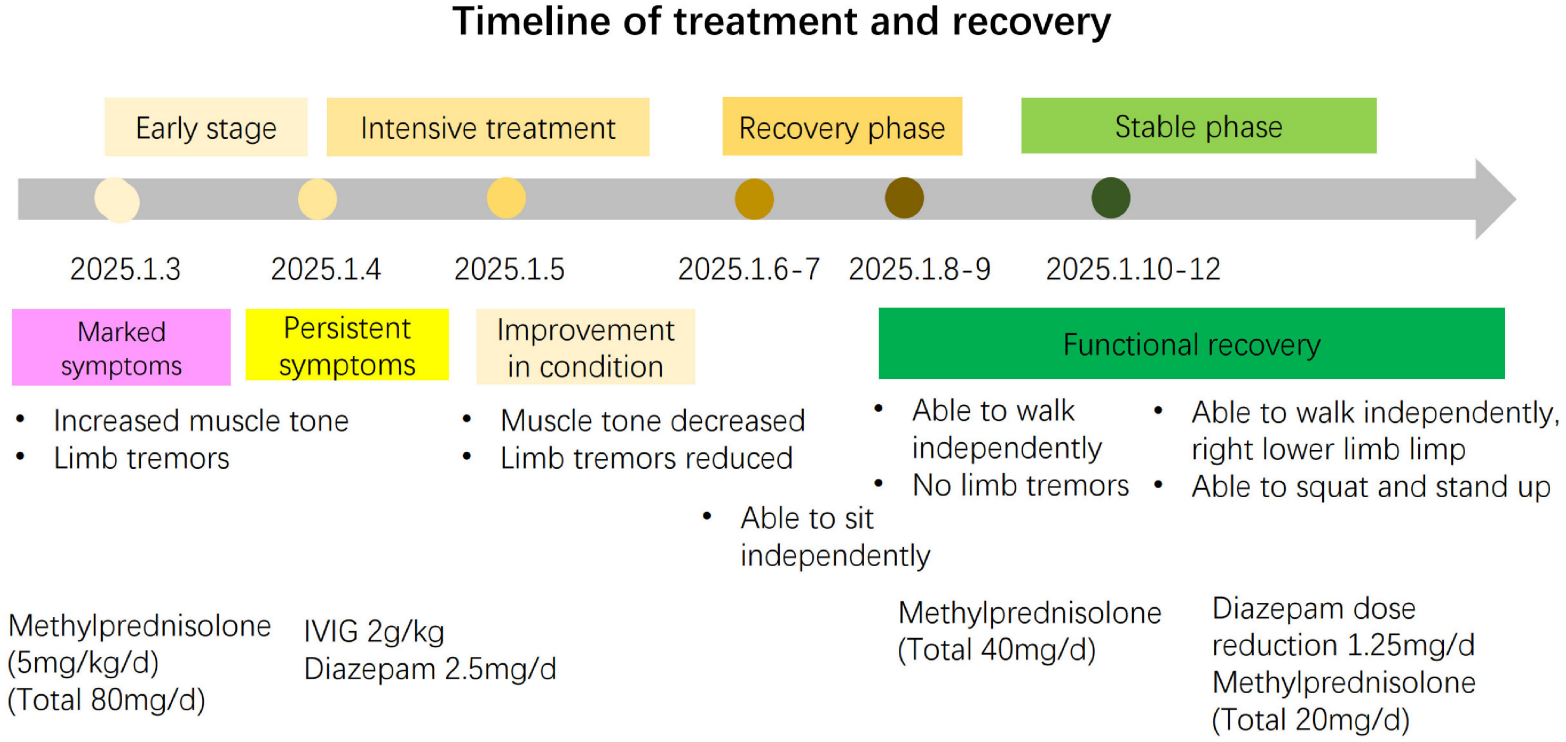
A brief timeline of the treatment and recovery process of the patient.

On the 3rd day of admission, the patient was treated with IVIG at a total dose of 2 g/kg in 3 days as well as corticosteroids (5 mg/kg/day intravenous methylprednisolone, followed by a tapered reduction) for 10 days with significant efficacy. Oral diazepam was administered (2.5mg/d). Oseltamivir for influenza virus infection was implemented. The patient had no neurological sequel at the time of discharge (**Figure 3**). Given the negative antibody results during the initial phase and complete clinical resolution at follow-up, antibody re-testing was not performed. All medications were discontinued one-month post-discharge, with the patient exhibiting normal motor function and attending regular kindergarten without restrictions.

## 3 Discussion

Chia et al. (5) in 2023 proposed possible diagnostic criteria for SPSD while highlighting the particular importance of electrophysiological diagnostic testing for the minority of seronegative SPSD cases. EMG is highly specific for the hallmark findings of sustained co-contraction of agonist and antagonist muscles, as well as continuous motor unit activity (CMUA), regardless of body region tested (6, 7). This phenomenon stems from the dysfunction of normal physiological GABAergic inhibitory mechanisms, resulting in continuous firing of gamma motor neurons due to deficiency of inhibitory signals, which leads to overstimulation of muscle spindles, clinically expressed as stiffness (8, 9). Following treatment with benzodiazepines, the motor unit potential may decrease or even disappear. In this patient, CMUA were observed in the antagonist muscle during agonist contraction (**Supplementary Video 1**). Unfortunately, simultaneous recording from the agonist muscle was not performed, and diazepam was not administered during the examination. However, subsequent administration of the medication in the ward showed marked alleviation. Future studies on similar cases should prioritize the use of dual-channel synchronous recording techniques to obtain more definitive evidence. Given the practical challenges of performing paraspinal EMG in pediatric patients, future cases could attempt to record paraspinal muscle activity using surface electrodes during electroencephalography (EEG) monitoring. According to previous studies, nearly all SPS patients exhibit spasmodic reflex myoclonus, manifested on EMG as reflexive repetitive electrical activity occurring 50-80ms after median or tibial nerve stimulation and lasting several seconds (10). This phenomenon further indicates motor unit hyperexcitability. Notably, ENG revealed repetitive compound muscle action potentials (R-CMAPs) after stimulation of the common peroneal and tibial nerves in this patient (**Figure 2B**).

Specific triggers for SPSD are still unknown, except for paraneoplastic associations. Although virus infections can trigger several autoimmune diseases, there is currently bare evidence supporting their involvement in SPSD. A case of GAD antibody-positive SPS was described following West Nile virus (WNV) infection (11). Dalakas et al. (12) reported a 39-year-old male patient who initially presented with mild stiff-leg syndrome (SLS) progressed to severe generalized SPS one week after the onset of COVID-19. Neo and colleagues (13)reported a case of SPS with cerebellar features in a 47-year-old female with SARS-CoV-2 infection. Furthermore, a case of PERM associated with GlyR antibodies has been described after SARS-CoV-2 infection (14). In a separate case, PERM manifestation occurred after varicella zoster virus (VZV) recurrence, accompanied by positive serum anti-GlyR antibodies. In our department, another pediatric case was observed, in whom symptoms of SPS emerged following mycoplasma infection, with a transiently positive anti-GlyR antibody in CSF, suggesting a potential post-infectious immune-mediated mechanism.

To our knowledge, this is the first H1N1-associated SPSD. Although the observed association does not necessarily imply causation, the temporal onset of SPSD symptoms, approximately one week after H1N1 infection, strongly suggests a virally triggered autoimmune or post-viral inflammatory process. The patient’s sustained response to IVIG further supports the hypothesis of H1N1-induced immune dysregulation. Unfortunately, convalescent serum samples were not collected. Consequently, it was not possible to provide more direct serological evidence to support the hypothesis that H1N1 infection triggers an autoimmune mechanism. This is one of the limitations of the study. In addition, anti-thyroid peroxidase antibodies (TPOAb) and anti-thyroglobulin antibodies (TGAb) should be tested despite normal thyroid function. The short interval between infection and neurological symptom onset may be attributed to a virus-triggered underlying autoimmune reaction, which could induce disease. This phenomenon has been documented in various autoimmune disorders following H1N1 infection, including immediately post-infectious GBS (15, 16), ADEM (17, 18), or acute necrotizing encephalopathy (ANE) (19).

We hypothesize this patient developed a post-infectious immune-mediated process predominantly affecting GABAergic neurons following acute H1N1 infection. The sequence or structural similarity between microbial antigenic epitopes may trigger an immune response, potentially leading to autoimmune diseases. This cross-reactivity and molecular mimicry can amplify specific immune responses, this possibility has been previously proposed in a case of SPS manifested after WNV infection, based on partial amino acid sequence homology between GAD65 and WNV (11). Concurrently, H1N1 viral infection may disrupt the blood-brain barrier (BBB), facilitating the entry of circulating autoantibodies or inflammatory cells into the central nervous system (CNS) (20). An infection-triggered immune response was considered a potential contributing factor, due to a favorable response to IVIG and glucocorticoid therapy, with significant symptom alleviation within a short period, confirming the presence of an immune-mediated mechanism in this case. However, further clinical observations and laboratory data are required to substantiate this hypothesis.

Currently, there is no specific treatment plan for SPSD. Among the etiological treatments, IVIG and steroid therapy are frequently attempted in clinical practice. Plasma exchange and rituximab are also commonly used. Symptomatic treatment aims to overcome the impairment of GABAergic neurotransmission to alleviate stiffness and spasms in patients. This may include benzodiazepines (such as diazepam or clonazepam), baclofen, as well as antiepileptic drugs that enhance GABA synthesis or facilitate GABAergic transmission. Clonidine has also been reported in the literature. A recent study described the successful use of clonidine in a patient with refractory stiff-person syndrome who presented with severe rigidity and painful spasms and did not respond to conventional symptomatic medications (21).

The natural course of SPSD typically exhibits slow progression. In this case, rapid clinical improvement was achieved following immunotherapy combined with diazepam, and no relapse was observed during the eight-month follow-up period. This favorable outcome may be attributed to early diagnosis and prompt immunotherapy. However, the possibility remains that the pathophysiology in such patients may differ from that of classical SPSD. Further clinical data and long-term follow-up studies are required to validate this hypothesis. For such patients, heightened vigilance must be maintained at the emergence of symptoms resembling SPSD following viral infection. Clinical management should prioritize early administration of antiviral agents and appropriate immunomodulators, accompanied by prompt EMG examinations, MRI, and antibody screening. It is particularly noteworthy that, given the prolonged duration required for autoantibody detection, empirical immunotherapy should be initiated as early as possible based on clinical manifestations after excluding other etiologies, with the aim of improving long-term prognosis in patients.

## Data Availability

The original contributions presented in the study are included in the article/**Supplementary Material**. Further inquiries can be directed to the corresponding authors.

## Supplementary material

Supplementary Video 1 Insertional activity was observed during the initial 1–4 seconds of needle electromyography (EMG) recording. From 5 to 15 seconds, continuous motor unit activity was recorded in the gastrocnemius muscle (antagonist) during voluntary contraction of the tibialis anterior muscle (agonist). After 16 seconds, while in the resting state, EMG activity with normal MUP morphology was still observed.
